# Benefit of a laparoscopic jejunostomy feeding catheter insertion to prevent bowel obstruction associated with feeding jejunostomy after esophagectomy

**DOI:** 10.1038/s41598-024-55020-w

**Published:** 2024-02-21

**Authors:** Hiroyuki Kitagawa, Keiichiro Yokota, Masato Utsunomiya, Tomoki Tanaka, Tsutomu Namikawa, Michiya Kobayashi, Satoru Seo

**Affiliations:** 1 Department of Surgery, Kochi Medical School, Kohasu-Okocho, Nankoku, Kochi 783-8505 Japan; 2 Department of Human Health and Medical Sciences, Kochi Medical School, Kohasu-Okocho, Nankoku, Kochi 783-8505 Japan

**Keywords:** Laparoscopic jejunostomy, Feeding catheter jejunostomy, Esophagectomy, Postoperative bowel obstruction, Esophageal cancer, Oesophageal cancer, Jejunum

## Abstract

The placement of a jejunostomy catheter during esophagectomy may cause postoperative bowel obstruction. The proximity of the jejunostomy site to the midline might be associated with bowel obstruction, and we have introduced laparoscopic jejunostomy (Lap-J) to reduce jejunostomy’s left lateral gap. We evaluated 92 patients who underwent esophagectomy for esophageal cancer between February 2013 and August 2022 to clarify the benefits of Lap-J compared to other methods. The patients were classified into two groups according to the method of feeding catheter insertion: jejunostomy via small laparotomy (J group, n = 75), and laparoscopic jejunostomy (Lap-J group, n = 17). Surgery for bowel obstruction associated with the feeding jejunostomy catheter (BOFJ) was performed on 11 in the J group. Comparing the J and Lap-J groups, the distance between the jejunostomy and midline was significantly longer in the Lap-J group (50 mm vs. 102 mm; P < 0.001). Regarding surgery for BOFJ, the distance between the jejunostomy and midline was significantly shorter in the surgery group than in the non-surgery group (43 mm vs. 52 mm; P = 0.049). During esophagectomy, Lap-J can prevent BOFJ by placing the jejunostomy site at the left lateral position to the midline and reducing the left lateral gap of the jejunostomy.

## Introduction

Esophagectomy is the basic treatment modality for esophageal cancer; however, postoperative anastomotic leakage is more common in esophageal cancer than in other gastrointestinal cancers^1^, and oral feeding may be delayed, during which time it is important to secure nutritional access routes for administration. Using feeding jejunostomy catheter during esophagectomy for esophageal cancer has been reported to be useful for early recovery and for home feeding after discharge^2–4^; however, as a complication, it can cause postoperative bowel obstruction associated with feeding jejunostomy (BOFJ)^5^. Therefore, some reports suggest it should be performed in selected cases with high perioperative risk rather than routinely in all patients^6,7^. However, it may be a useful nutrition access route when postoperative anastomotic leakage occurs.

We previously reported that a shorter distance between the midline of the abdomen and feeding jejunostomy through a small laparotomy wound increases the risk of BOFJ^8^. As a countermeasure, we changed the nutrition access route from jejunostomy to duodenostomy through the round ligament of the liver^9–11^ in 2018. However, we encountered a case of intra-abdominal abscess caused by a leak at the tube-entry site. We considered the cause to be the longer distance between the abdominal wall and the duodenal tube entry point in the posterior mediastinal route reconstruction compared to the retrosternal route, which increases the risk of leakage even when covered with a round ligament. Therefore, in December 2021, we introduced a gastroduodenal feeding catheter in cases of retrosternal route reconstruction, and laparoscopic jejunostomy (Lap-J) in cases of posterior mediastinal route reconstruction.

The purpose of Lap-J is to move the jejunostomy site away from the midline of the abdomen and reduce the gap on the left lateral side. This study retrospectively reviewed the results of these feeding catheter insertion methods and revealed the benefits of Lap-J in preventing BOFJ.

## Patients and methods

We reviewed 92 patients who underwent thoracoscopic esophagectomy for esophageal cancer with gastric tube reconstruction between February 2013 and August 2022 and divided them into two groups according to the method of feeding catheter insertion: jejunostomy via a small open laparotomy (J group) and laparoscopic jejunostomy (Lap-J group). Patient background, surgical outcomes, postoperative complications, and bowel obstruction were retrospectively reviewed.

This study was conducted in accordance with the Declaration of Helsinki, Ethical Guidelines for Life Sciences and Medical Research Involving Human Subjects, and other relevant guidelines and considered the human rights and safety of the research participants. Informed consent was waived, and approval was obtained from the Ethics Committee of Kochi Medical School (ERB-104871) to disclose information on this study as an opt-out on our hospital website.

### Procedure

After thoracoscopic esophagectomy and mediastinal lymph node dissection, patients were transferred to the supine position and underwent laparoscopic gastric mobilization and dissection of the lesser curvature lymph nodes. Laparotomy was performed in patients with a history of laparotomy. After a small laparotomy, the blood flow in the stomach was evaluated using indocyanine green, a near-infrared fluorescent contrast agent, to create a gastric tube^12^, elevated to the neck and anastomosed to the cervical esophagus, after which a feeding catheter was inserted.

### Needle catheter jejunostomy

Approximately 30 cm from the anal side of the ligament of Treitz, a purse-string suture was made on the jejunal wall with an absorbable thread, and a 9 Fr tube (Kangaroo Jejunostomy Catheter, Covidien Japan, Tokyo, Japan) was inserted into the jejunum using the Seldinger’s technique. After approximately 25 cm of insertion, the tube was fixed with a purse-string suture, and three additional sutures were placed using the Witzel procedure^8^. The tube was passed out of the abdominal wall using the Seldinger’s technique and fixed with the abdominal wall using a non-absorbable thread longitudinally for at least 5 cm.

### Laparoscopic needle catheter jejunostomy

**Figure 1:**
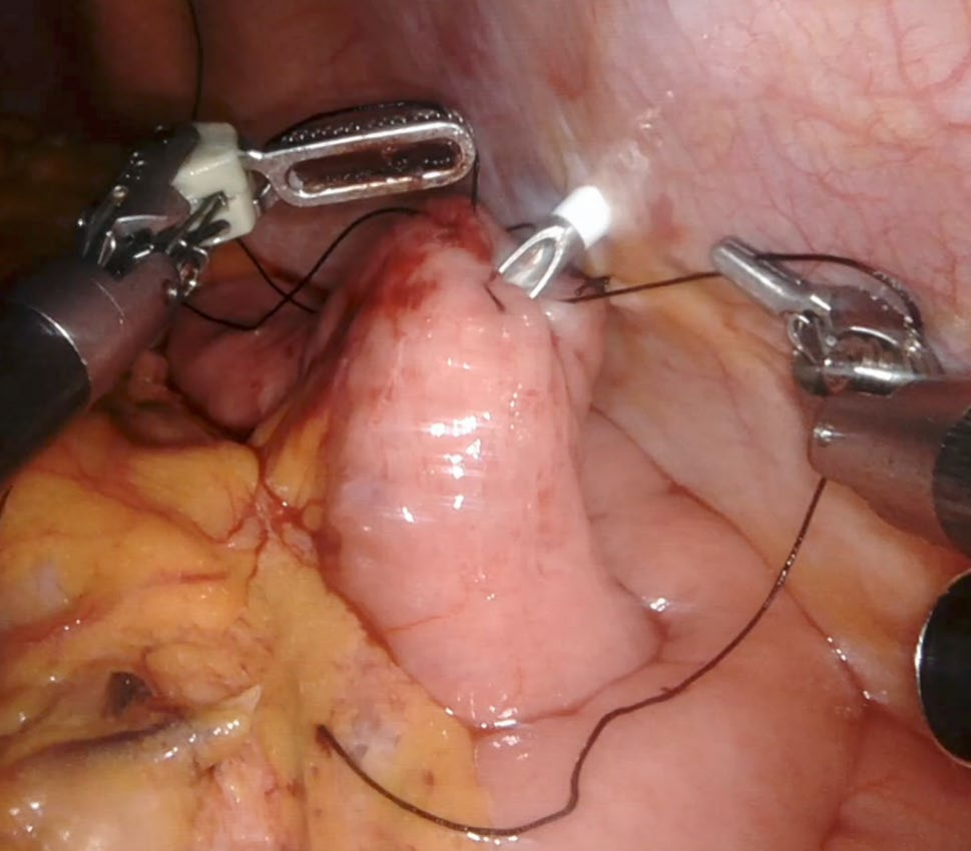
A 9 Fr tube was inserted into the jejunum by puncturing the abdominal wall, and purse-string sutures were made from the outside using a Seldinger kit.

**Figure 2:**
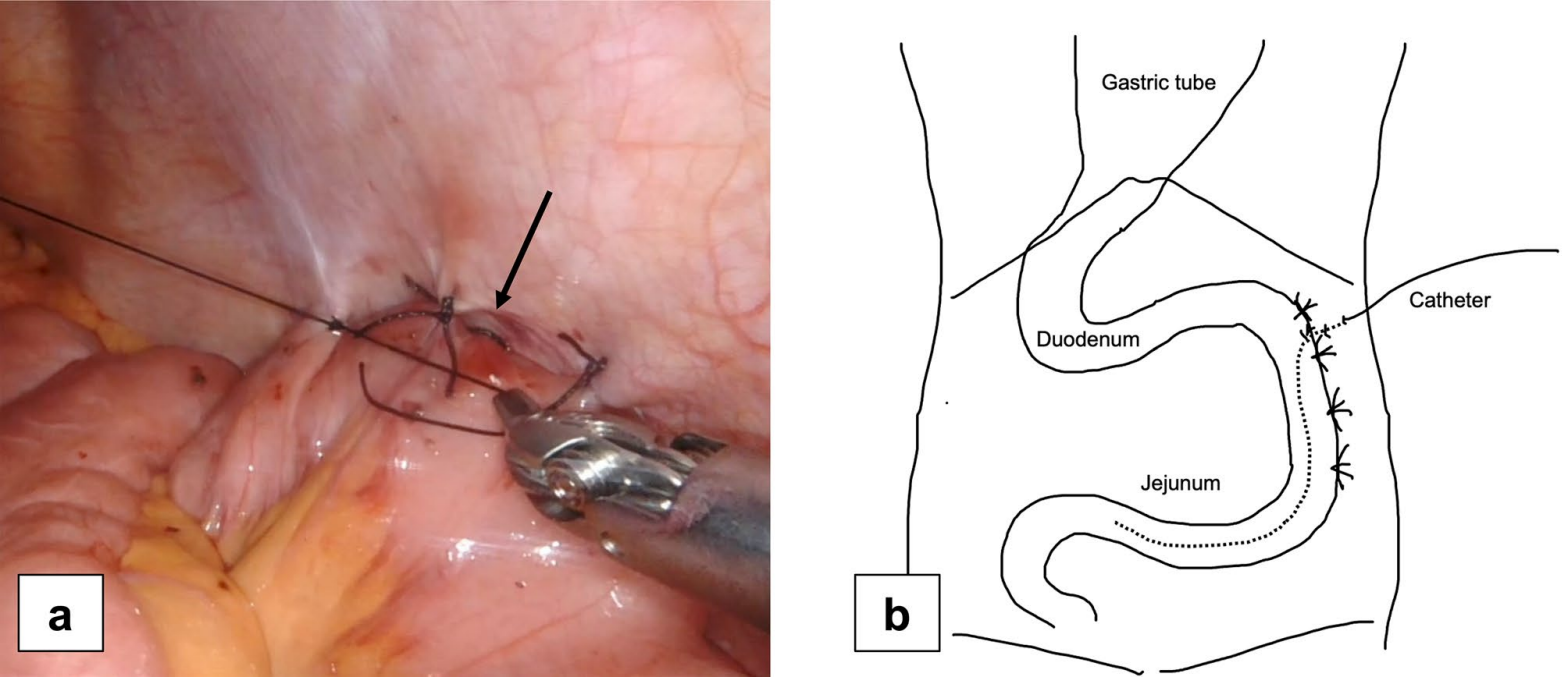
(**a**) After longitudinal fixation of the jejunum, an additional fixation was made at the oral side of the tube insertion point (arrow) to prevent bending. (**b**) Schema of a laparoscopic jejunostomy feeding catheter insertion.

Laparoscopically, a purse-string suture was made on the jejunal wall approximately 30 cm from the ligament of Treitz, and one fixation was made on the ventral abdominal wall of the descending colon. A 9 Fr tube was inserted approximately 30 cm into the jejunum by puncturing the abdominal wall, and purse-string sutures were made from the outside using a Seldinger kit (Fig. 1). Saline was injected, and the tube was inserted in a floating motion. The outer tube was then removed and fixed using a purse-string suture. In order to prevent torsion, the anal side of the jejunum was fixed to the abdominal wall longitudinally by at least 5 cm using a non-absorbable suture. Additional fixation was performed on the oral side of the tube insertion point (Fig. 2a,b) to prevent bending.

### Postoperative enteral nutrition

The patient was admitted to the ICU postoperatively and weaned from the ventilator the day after surgery. Enteral nutrition was started on the morning of the day after surgery at 20 ml/h and titrated to 40 ml/h by postoperative day 7. Doses were increased to meet nutritional requirements in patients who developed anastomotic leakage and maintained or decreased in those who did not and could consume food orally. The tube was left in place, and the patient was discharged from the hospital with instructions to allow enteral feeding. The amount and method of feeding were determined at the discretion of each patient. The tube was removed after oral intake was checked at an outpatient visit approximately 2 months after surgery.

### Outcome parameters

Patient characteristics included age, sex, cancer histology, TNM classification eighth clinical stage^13^, weight and body mass index immediately before surgery, presence or absence of laparoscopic gastric mobilization, reconstruction route, anastomosis method, feeding catheter construction method, operation time and blood loss, postoperative complications such as recurrent pneumonia, anastomotic leakage, presence of wound infection, and postoperative hospital stay. For feeding jejunostomy, the distance between the jejunostomy site and the midline, duration until removal of the feeding catheter, the presence or absence of BOFJ, and weight changes at 3, 6, and 12 months postoperatively were recorded, and the ratio to preoperative weight was examined.

**Figure 3:**
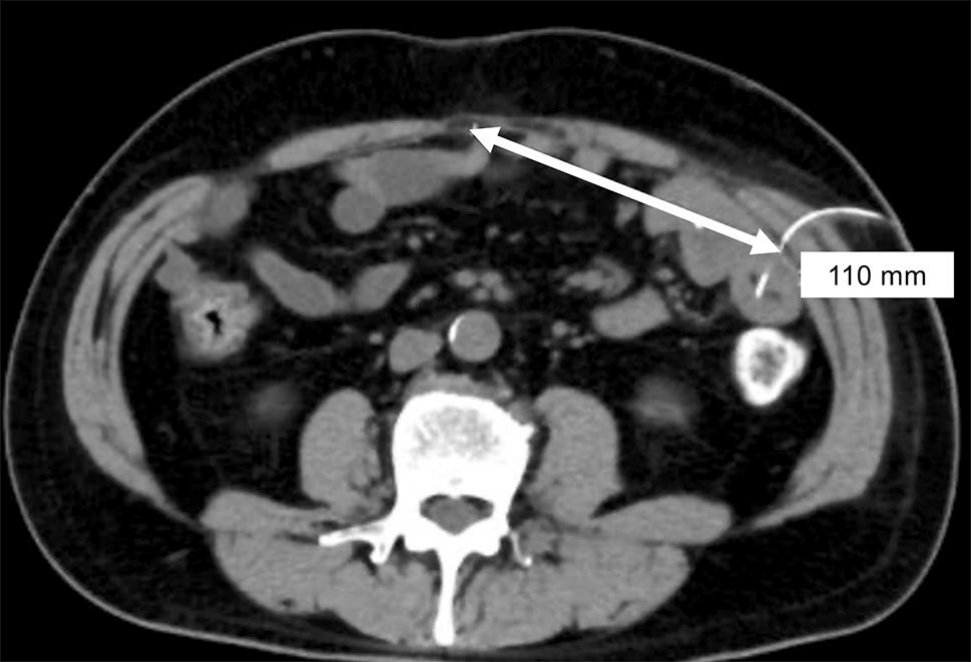
The linear distance between the jejunostomy site and the midline of the abdomen was measured on the CT image.

The distance between the jejunostomy site and the midline of the abdomen was measured on 5 mm slice thickness (Fig. 3). The Mann–Whitney U test was used to evaluate differences in continuous variables, and Pearson’s chi-squared test was used for categorical variables. All analyses were performed using JMP 13 (SAS Institute Inc., Cary, NC, USA), with statistical significance set at P < 0.05.

## Results

**Figure 4:**
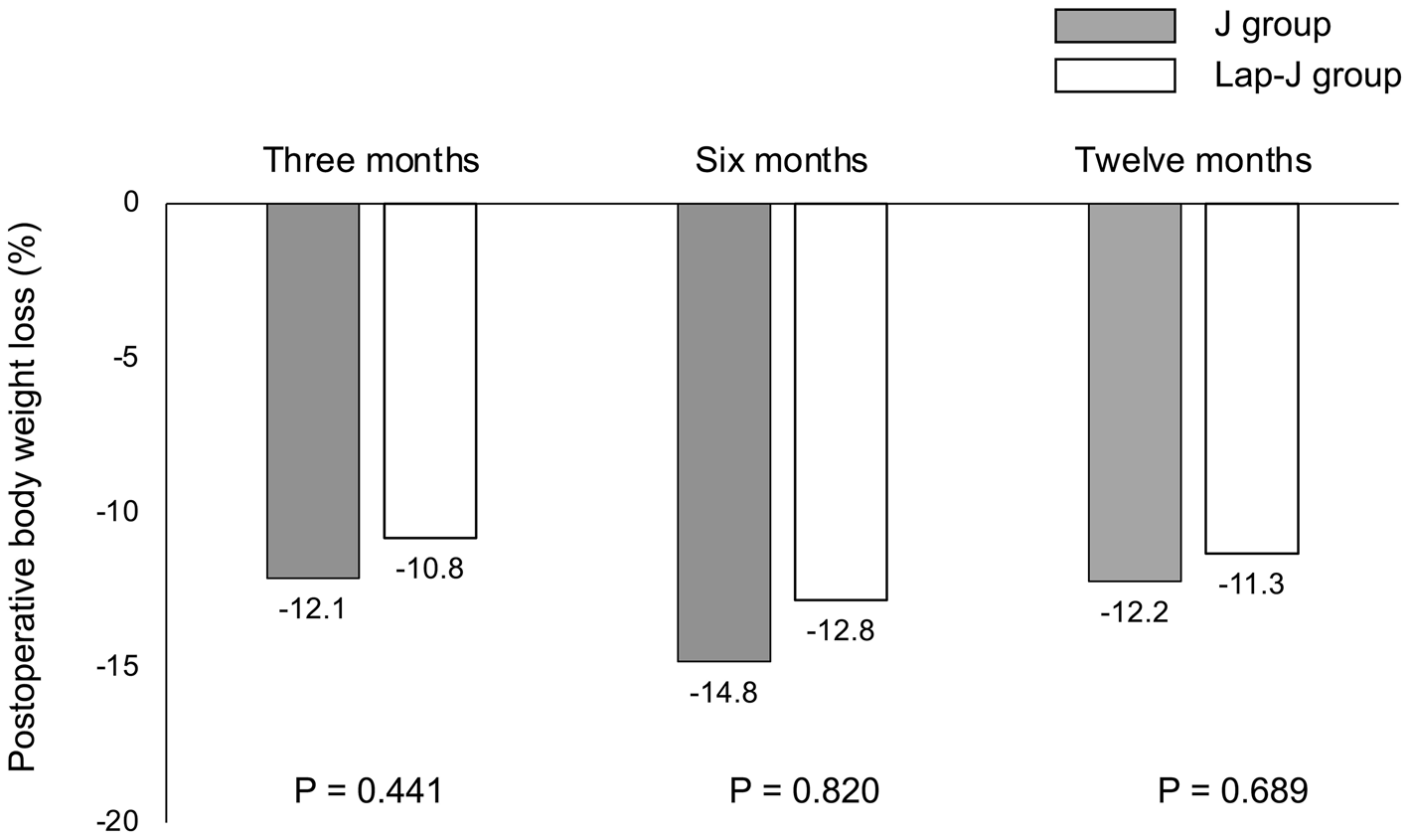
Comparison of postoperative body weight change between the J and Lap-J groups.

Table 1 shows a characteristic of the 92 patients who underwent the thoracoscopic esophagectomy with feeding jejunostomy catheter during esophagectomy for esophageal cancer and comparison between the two groups. Posterior mediastinal route reconstruction was common after laparoscopic gastric mobilization in both groups, however robotic assisted surgery was included only in the Lap-J group. There was no significant difference in operative time; however, the Lap-J group had significantly lower blood loss volume (160 mL vs. 100 mL; P = 0.007), longer jejunostomy creating time (20 min vs. 37 min; P = 0.007), lower incidence of recurrent laryngeal nerve palsy (25.3% vs. 11.8%; P = 0.007), and longer distance between the jejunostomy site and the midline (50 mm vs. 102 mm; P < 0.001). The time to remove the feeding catheter was longer in the J group; however, the difference was not significant. Surgery for the BOFJ was performed on 11 patients in the J group. Of these, seven were emergency surgeries for strangulation, and four were elective surgeries due to abdominal pain symptoms and a whir sign suggestive of torsion on CT. The rate of postoperative weight loss was also not significantly different between the two groups (Fig. 4).

**Table 1:** Characteristics of the 92 patients who underwent the thoracoscopic esophagectomy with feeding jejunostomy catheter during esophagectomy for esophageal cancer and comparison between the two groups.

	All patients n = 92	J group n = 75	Lap-J group n = 17	P value
Sex, male, n (%)	85 (92.4)	60 (80.0)	15 (88.2)	0.730
Age, years	68 (62–90)	68 (43–82)	71 (46–90)	0.307
Histology, squamous cell carcinoma, n (%)	81 (88.0)	66 (88.0)	15 (88.2)	0.266
Stage I/II/III/IV, n (%)	28/16/25/23 (30.4/17.4/27.2 /25.0)	22/12/22/19 (29.3/16.0/29.3/25.3)	6/4/3/4 (35.3/25.5/17.7/23.5)	0.329
Preoperative body weight (kg)	55.8 (36.0–81.0)	55.0 (39.9–78.0)	63.8 (36.0–81.0)	0.184
Preoperative body mass index (kg/m^2^)	21.3 (14.4–33.1)	20.9 (15.1–30.0)	24.1 (14.4–33.1)	0.066
Laparoscopic gastric mobilization, n (%)	74 (80.4)	59 (78.7)	15 (88.2)	0.470
Robotic assisted, n (%)	7 (7.6)	0	7 (41.2)	< 0.001
Reconstruction route				0.565
Posterior mediastinum, n (%)	88 (95.7)	72 (96.0)	16 (94.1)	
Retrosternal, n (%)	4 (4.3)	3 (4.0)	1 (5.9)	
Anastomosis, circular stapler/hand-sewn, n (%)	88/4 (95.7/4.3)	71/4 (94.7/5.3)	17/0 (100/0)	> 0.999
Operative time (min)	611 (456–859)	613 (456–859)	600 (521–748)	0.601
Blood loss (mL)	150 (50–1600)	160 (50–1600)	100 (50–250)	0.007
Jejunostomy creating time (min)	25 (15–59)	20 (15–23)	37 (18–59)	0.007
Complications				
Recurrent laryngeal nerve palsy, n (%)	21 (22.8)	19 (25.3)	2 (11.8)	0.007
Pneumonia, n (%)	10 (10.9)	9 (12.0)	1 (5.9)	0.682
Anastomotic leakage, n (%)	9 (9.8)	8 (10.7)	1 (5.9)	> 0.999
Wound infection, n (%)	19 (20.7)	15 (20.0)	4 (23.5)	0.746
Hospital stay (days)	20 (10–138)	20 (10–138)	22 (14–64)	0.302
Distance between the site of jejunostomy and midline (mm)	51 (22–133)	50 (22–76)	102 (68–133)	< 0.001
Duration until feeding catheter removal (days)	73 (5–316)	78 (6–316)	56 (5–127)	0.174
Surgery for bowel obstruction associated with feeding catheter, n (%)	11 (12.0)	11 (14.7)	0	0.091

Values represent median (range).

**Figure 5:**
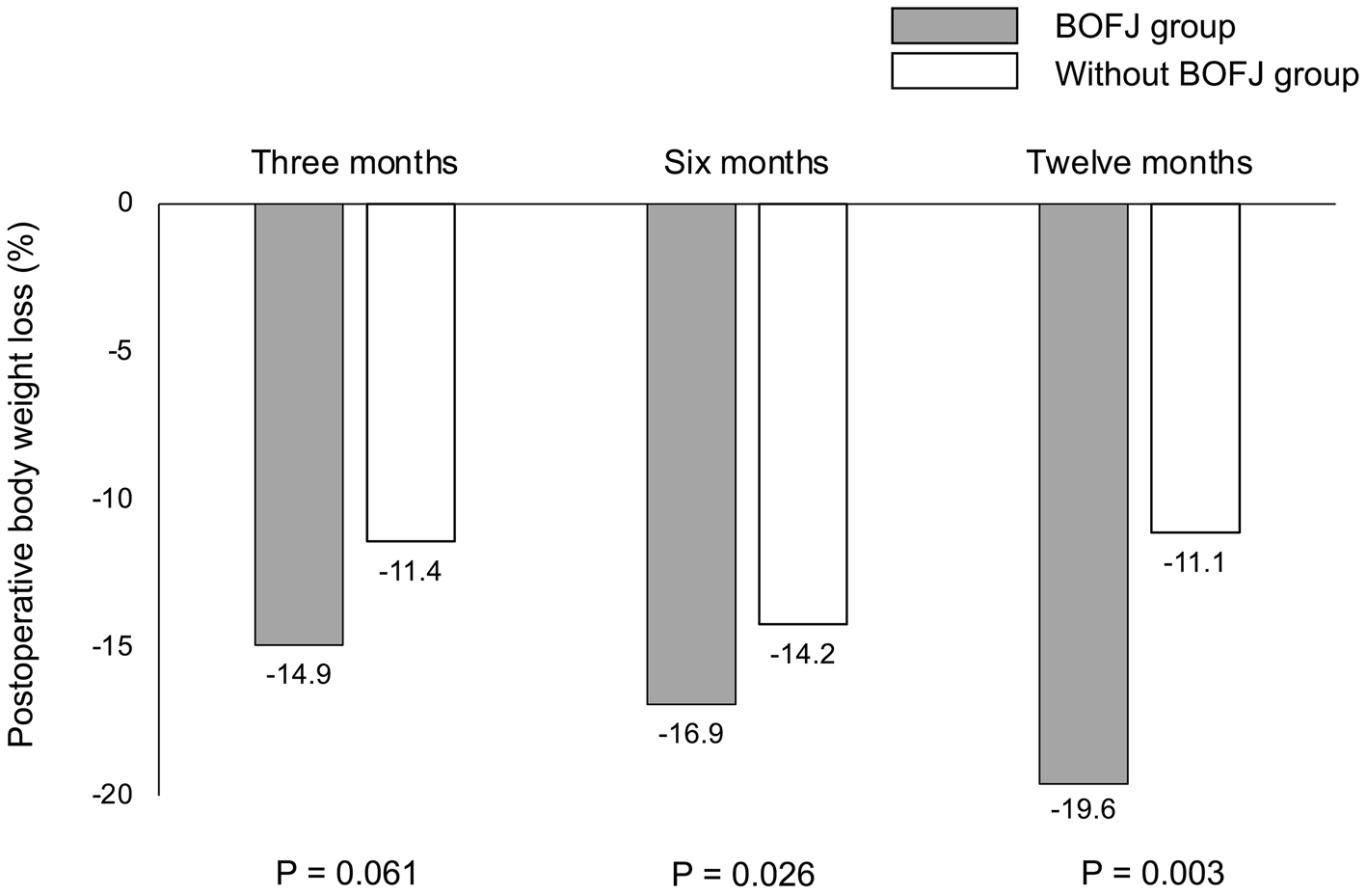
Comparison of postoperative body weight change between with or without surgery for bowel obstruction associated with feeding jejunostomy catheter. The postoperative body weight rate in the BOFJ group was significantly decreased than in the non-BOFJ group at 6 and 12 months after the esophagectomy.

When compared with and without surgery for BOFJ (Table 2), the BOFJ group had significantly shorter distance between the jejunostomy site and midline (43 mm vs. 52 mm; P = 0.049), and their postoperative body weight rate was significantly decreased at 6 (83.1% vs. 85.8%; P = 0.026), and 12 months (80.4% vs. 88.9%; P = 0.003) after the esophagectomy (Fig. 5).

**Table 2:** Comparison between the surgery for bowel obstruction associated with feeding catheter or not.

	Surgery for bowel obstruction associated with feeding catheter		
	Yes (n = 11)	No (n = 81)	P value
Sex, male, n (%)	8 (72.7)	67 (82.7)	0.420
Age, years	64 (52–75)	68 (43–90)	0.173
Histology, squamous cell carcinoma, n (%)	9 (81.8)	72 (88.9)	0.260
Stage I/II/III/IV, n (%)	5/3/1/2 (45.5/27.3/9.1/18.2)	23/13/24/21 (28.4/16.1/29.3/25.9)	0.473
Preoperative body weight (kg)	57.0 (43.1–75.9)	55.6 (36.0–81.0)	0.367
Preoperative body mass index (kg/m^2^)	22.9 (18.6–26.0)	21.2 (14.4–33.1)	0.741
Laparoscopic procedure, n (%)	11 (100)	63 (77.8)	0.114
Robotic assisted, n (%)	0	7 (8.6)	0.593
Reconstruction route			> 0.999
Posterior mediastinum, n (%)	11 (100)	77 (95.1)	
Retrosternal, n (%)	0	4 (4.9)	
Anastomosis, circular stapler/hand-sewn, n (%)	10/1 (90.9/9.1)	78/3 (96.3/3.7)	0.405
Operative time (min)	611 (456–772)	612 (473–859)	0.782
Blood loss (mL)	170 (50–490)	150 (50–1600)	0.866
Complications			
Recurrent laryngeal nerve palsy, n (%)	3 (27.3)	18 (22.2)	0.824
Pneumonia, n (%)	1 (9.1)	9 (11.1)	> 0.999
Anastomotic leakage, n (%)	1 (9.1)	8 (9.9)	> 0.999
Wound infection, n (%)	3 (27.3)	16 (19.8)	0.691
Hospital stay (days)	17 (13–55)	20 (10–138)	0.731
Method of feeding catheter			0.207
Jejunostomy, n (%)	11 (100)	64 (79.0)	
Laparoscopic jejunostomy, n (%)	0	17 (21.0)	
Distance between the site of jejunostomy and midline (mm)	43 (22–63)	52 (26–133)	0.049
Duration until feeding catheter removal (days)	55 (6–184)	77 (5–316)	0.424

Values represent median (range).

## Discussion

This study demonstrated that the Lap-J group had a longer distance between the jejunostomy site and the midline and fewer BOFJ compared to the J group. BOFJ is caused by the small intestine torsion of the anal side over the jejunostomy into the gap between the abdominal wall fixation sites. Kamada et al.^14^ reported that the vertical distance between the jejunostomy and navel was associated with BOFJ. We also reported that the proximity of the jejunostomy to the midline of the abdomen is a risk factor for BOFJ^8^ and that the risk of torsion due to internal herniation into the gap and strangulation may increase depending on the position of the jejunostomy site. In contrast, Shiraishi^15^ proposed a novel laparoscopic feeding jejunostomy technique, the “curtain method,” which involves the closure of the triangular zone. In this study, Lap-J prevented BOFJ by reducing the left lateral gap.

As previously reported^8^, the postoperative weight loss was more influenced by the presence or absence of surgery for BOFJ than by the difference in catheter insertion procedures. In our previous report, we considered that postoperative weight loss may have led to a larger gap in the bowel due to fat and muscle atrophy, which may have led to more volvulus in laparoscopic surgery with fewer adhesions, as surgery cases tended to have a higher preoperative BMI and more laparoscopic cases; however, this was not the case in this study. Therefore, rather than weight loss being a factor in bowel torsion, weight loss may result from reduced food intake due to impaired food passage and abdominal symptoms caused by bowel torsion. Thus, prevention of BOFJ was important for reducing postoperative weight loss.

A gastroduodenal feeding catheter is useful for avoiding torsion because the horizontal duodenal limb on the anal side is fixed to the retroperitoneum, reducing the risk of bowel obstruction compared with jejunostomy^9–11,16^ then we have introduced since 2018. In cases of retrosternal route reconstruction, the stomach and duodenum are close to the abdominal wall, which makes the construction technique easy and allows sufficient coverage with hepatic round ligament fat. In cases of posterior mediastinal route reconstruction, the distance between the tube entry site and the abdominal wall is long, even with Kocher’s mobilization, and coverage with hepatic round ligament fat is insufficient. Therefore, we decided to select a method of inserting the feeding catheter according to the reconstruction route and selected a gastroduodenal feeding catheter in cases of retrosternal reconstruction and a jejunostomy in cases of retro mediastinal reconstruction. In the case of jejunostomy, we decided to perform it laparoscopically as we considered it important to reduce the risk of torsion by reducing the gap on the left side of the abdominal wall fixation by creating a jejunostomy on the left lateral side at a distance between the jejunostomy site and the midline, difficult to achieve via a small open laparotomy incision. As a result, the Lap-J group had a longer distance between the jejunostomy site and midline than the J and no-BOFJ groups.

A limitation of this study was the small retrospective sample size, which may have led to a bias towards more BOFJ in the J group with a longer observation period than in the Lap-J group. However, the median time between esophagectomy and surgery for BOFJ was 197 days, approximately 6 months after surgery; therefore, the Lap-J group was considered to have had sufficient observation time for BOFJ. Another limitation was that the length of the feeding tube fixed to the abdominal wall was not measured. Longer fixation along the long axis from the enterostomy site might have prevented bowel obstruction, even if the jejunostomy site was close to the midline of the abdomen^17^. Regarding this, we consider the length of fixation to the abdominal wall sufficient to prevent torsion, as it is generally 5 cm. However, in the future, attention should be paid to the length of fixation to the abdominal wall.

In conclusion, during esophagectomy, Lap-J could prevent BOFJ by placing the jejunostomy site at the left lateral position to the midline and reducing the left lateral abdominal gap, compared with jejunostomy via a small laparotomy. We need to further investigation about Lap-J, including robotic surgery, which may be more maneuverable than laparoscopy.

## Data Availability

The datasets generated during and/or analyzed during the current study are available from the corresponding author on reasonable request.
